# Intraoperative hypotension - a neglected causative factor in hospital-acquired acute kidney injury; a Mayo Clinic Health System experience revisited

**DOI:** 10.12861/jrip.2015.13

**Published:** 2015-09-01

**Authors:** Macaulay Amechi Chukwukadibia Onuigbo, Nneoma Agbasi

**Affiliations:** ^1^Mayo Clinic College of Medicine, Rochester, MN 55905, USA; ^2^Department of Nephrology, Mayo Clinic Health System, Eau Claire, USA; ^3^North East London NHS Foundation Trust, UK

**Keywords:** Acute kidney injury, Chronic kidney disease, Estimated glomerular filtration rate, Renal replacement therapy, Serum creatinine trajectory

## Abstract

Acute kidney injury (AKI) is a relatively common complication of cardiothoracic surgery and has both short- and long-term survival implications, even when AKI does not progress to severe renal failure. Given that currently, there are no active effective treatments for AKI, other than renal replacement therapy when indicated, the focus of clinicians ought to be on prevention and risk factor management. In the AKI-surgery literature, there exists this general consensus that intraoperative hypotension (IH) following hypotensive anesthesia (HA) or controlled hypotension (CH) in the operating room has no significant short-term and long-term impacts on renal function. In this review, we examine the basis for this consensus, exposing some of the flaws of the clinical study data upon which this prevailing consensus is based. We then describe our experiences in the last decade at the Mayo Clinic Health System, Eau Claire, in Northwestern Wisconsin, USA, with two selected case presentations to highlight the contribution of IH as a potent yet preventable cause of post-operative AKI. We further highlight the causative although neglected role of IH in precipitating postoperative AKI in chronic kidney disease (CKD) patients. We show additional risk factors associated with this syndrome and further make a strong case for the elimination of IH as an achievable mechanism to reduce overall, the incidence of hospital acquired AKI. We finally posit that as the old saying goes, prevention is indeed better than cure.

**Implication for health policy/practice/research/medical education:**

Acute kidney injury (AKI) is a relatively common complication of cardiothoracic surgery and has both short- and long-term survival implications, even when AKI does not progress to severe renal failure. Given that currently, there are no active effective treatments for AKI, other than renal replacement therapy when indicated, the focus of clinicians ought to be on prevention and risk factor management. In the AKI-surgery literature, there exists this general consensus that intraoperative hypotension (IH) following hypotensive anesthesia (HA) or controlled hypotension (CH) in the operating room has no significant short-term and long-term impacts on renal function.

## Introduction

### Currently identifiable risk factors for hospital acquired acute kidney injury

The just published Atherosclerosis Risk in Communities Study assessed the risk factors for hospital acquired acute kidney injury (AKI) in 11 011 participants followed from 1996-1998 (baseline) through to 2010 (1). Among the various recognized risk factors adjusted for in the multivariate Cox regression analysis in this large study were age, sex, race, eGFR (modeled as a linear spline with a knot at 60 ml/min per 1.73 m^2^), log albumin creatinine ratio (ACR), hypertension, diabetes, coronary heart disease, use of diuretics, allopurinol, and losartan (1). Additionally, following this analysis, a new risk factor for hospital acquired AKI, baseline plasma urate >5.0 mg/dl, was identified as an independently associated risk factor for hospitalized AKI (1). Furthermore, a recent analysis of factors associated with hospital-acquired AKI following cardiac surgery enumerated the following as identifiable risk factors – older age, higher preoperative creatinine, higher plasma glucose level, lower left ventricular ejection fraction, high preoperative blood urea nitrogen (>20 mg/dL), high creatinine level (>1 mg/dL), high uric acid (>7 mg/dL) and lower albumin (<4 g/dL) or lower intraoperative hemoglobin (<8 g/dL) had a higher risk for postoperative AKI (2). Conspicuously absent from these listed identifiable causes of hospital acquired AKI is intraoperative hypotension (IH).

### Intraoperative hypotension and acute kidney injury in the literature

Up until now, the prevailing consensus in the literature is that IH, the result of hypotensive anesthesia (HA) or controlled hypotension (CH), has no deleterious effects on the cardiovascular system and kidney function (3-13). Choi and Samman completed an extensive systematic review of 833 potentially relevant articles, and selected 314 articles for analysis including randomized clinical trials, controlled clinical trials, case-controlled studies, prospective case series and retrospective case series (3). Choi and Samman demonstrated after this very meticulous and extensive review of the surgical AKI literature that the benefits of HA were significant decrease of blood loss, significant decrease in blood transfusion rate, improved surgical field and significant reduction in operation time (3). In terms of the risks of HA, no significant changes in cerebral, cardiovascular, renal and hepatic functions in patients receiving HA compared to control were demonstrable (3-13).

Thompson et al (13) in a brilliant case series of 30 patients attempted to determine whether HA or the method of inducing hypotension has any effect on postoperative brain, liver, or kidney function and myocardial status following total hip arthroplasty. Anesthesia was achieved with halothane-nitrous oxide for total hip arthroplasty and the 30 patients were randomly assigned to one of 3 groups. In 2 groups mean arterial blood pressure (MABP) was decreased to 50 mm Hg by high inspired concentrations of halothane (n = 90) or sodium nitroprusside (n = 12)(13). In the third group (n = 9) MABP was maintained within 20% of control. Intraoperative blood losses decreased from 1.183 +/- 172 ml in the normotensive group to 406 +/- 102 ml and 326 +/- 41 ml in the halothane and nitroprusside hypotensive groups, respectively. Neither method of inducing hypotension nor hypertensive technique affected the results of postoperative tests of cerebral, hepatic, or renal function and myocardial status (13). These tests were performed before anesthesia and operation and at intervals in the postoperative course. Thompson et al (13) therefore concluded that in this small group of patients, deliberate hypotension for total hip arthroplasty added no morbidity and significantly shortened operating time, decreased blood loss, and decreased the number of blood transfusions needed. However, it must be acknowledged that this study by Thompson et al, excluded all patients with cerebrovascular accident (CVA) or hypertension, all patients with renal disease as indicated by an increased serum creatinine level, and the mean age range in this study was 57-61 years. Thompson et al, (13) further noted that in this study, blood and urinary chemistry including creatinine clearance studies reflected no impairment of renal function after 2 days. The authors however recalled that whether a longer hypotensive period would produce injury to the kidneys remained unanswered by their study.

Moreover, in the more recent nephrology literature, several studies of the effect of CH in patients undergoing laparoscopic or robot-assisted partial nephrectomies have also concluded that CH did not show any detrimental impact on renal function during and after laparoscopic and robot-assisted partial nephrectomy (14-16). Nevertheless, and most importantly, the patients in these partial nephrectomy studies were generally younger, mean age <60 years, without preexisting renal disease with serum creatinine mostly ≤1 mg/dL, and with eGFR in the 75-90 ml/min/1.73 sq. m BSA range (14-16). Additionally, the studies excluded patients with poorly controlled or uncontrolled hypertension (14-16). Also, MABP achieved during CH in these studies was 65 mm Hg, and mean duration of CH was relatively short, ranging from 10 minutes to 50 minutes (14-16). Besides, the median operation time was 2 hours (1-3.5) (15).

From the foregoing, although postoperative AKI is a well-studied phenomenon in the literature, the AKI literature has continued to not recognize IH as a significant pathogenetic factor in the causation of postoperative hospital acquired AKI (1-16).

Contrary to the preceding sentiments, our experiences at the Mayo Clinic Health System Renal Unit in Northwestern Wisconsin, USA, would strongly argue that IH is a potent albeit preventable causative factor in producing postoperative AKI, in both cardiac and noncardiac surgery. First, we would discuss renal autoregulation and hypotension in the older chronic kidney disease (CKD) patient, describe 2 selected case presentations, and then conclude with our hypotheses regarding this titillating phenomenon of IH as a potent albeit preventable risk factor for postoperative AKI in hospitalized patients.

### Renal autoregulation, hypotension and the older patient with chronic kidney disease

In a recent 2015 review, we had discussed the concept of nephroprevention in the oldest old with chronic kidney disease (17). Clearly, the older (>65 year old) patient with chronic kidney disease is encumbered with such comorbidities as renal senescence, polypharmacy with increased likelihood of exposure to potentially nephrotoxic agents such as non-steroidal anti-inflammatory drugs (NSAIDs) and angiotensin inhibitors, hypertension, renovascular disease and other maladies (17-22). Although there remain many unanswered questions and controversies regarding the control of renal autoregulation and glomerulo-tubular balance both in health and in disease (20), evidence demonstrate that many of the renal auto-regulatory mechanisms are derelict in the older (>65 year old) and more so in the older patient with more advanced CKD (17-22).

### Case presentations

In early 2013, we had described a new syndrome of accelerated postoperative AKI-on-CKD occurring in previously stable CKD patients who at the time of surgery were on triple whammy medications (23-26).We shall present here a case of quadruple whammy and will demonstrate the extent of IH that he experienced prior to exhibiting the accelerated postoperative AKI. We shall describe here one of the 2 case presentations from the Spring of 2013 and a second more recent case presentation from 2014 to highlight and show case the implication of IH in the causation of postoperative hospital acquired AKI.

### Case I

**Figure 1:**
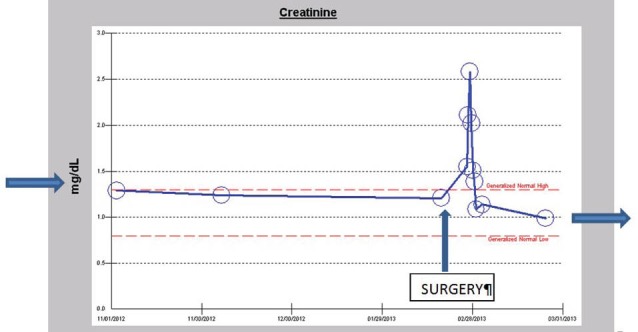
Serum creatinine trajectory showing rapidly rising serum creatinine following intraoperative hypotension (IH) during right hip arthroplasty.

**Figure 2:**
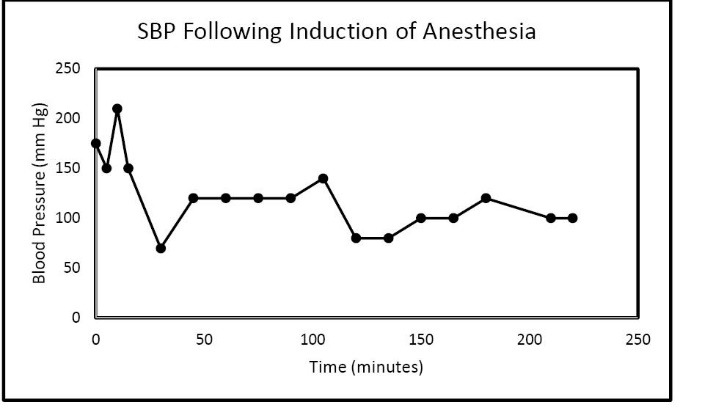
Significant intraoperative hypotension (IH) during anesthesia for right hip arthroplasty as reflected by continuous systolic blood pressure (SBP) recordings.

**Figure 3:**
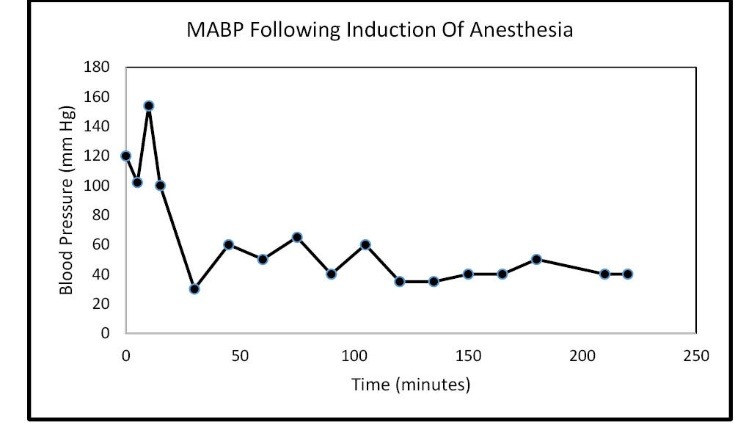
Significant intraoperative hypotension (IH) during anesthesia for right hip arthroplasty as reflected by continuous mean arterial blood pressure (MABP) recordings.

**Figure 4:**
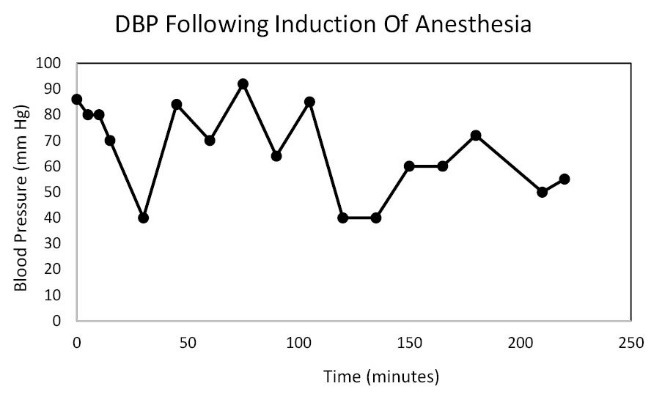
Significant periods of intraoperative hypotension (IH) during anesthesia for right hip arthroplasty as reflected by continuous diastolic blood pressure (DBP) recordings.

In the Spring of 2013, a 46-year-old morbidly obese (170 kg) hypertensive Caucasian male patient, a current smoker, otherwise stable stage III CKD, serum creatinine of 1.21 mg/dL, eGFR of 70 ml/min/1.73 sq m. BSA developed AKI following an otherwise uneventful elective right hip arthroplasty for symptomatic degenerative joint disease (23-26). Outpatient medications were Lisinopril 40 mg daily and Hydrochlorothiazide 25 mg daily. Besides, he received a preoperative dose of Celecoxib (Celebrex^®^), a Cox II Inhibitor, per “operative analgesia protocol,” thus completing the ‘triple whammy circle.’ Rapidly, within 36 hours after the surgical operation, he had developed oliguric AKI on CKD, complicated with metabolic acidosis. His serum creatinine had more than doubled to 2.58 mg/dL, eGFR of 28 ml/min/1.73 sq m. BSA (Figure 1). A critical and meticulous review of his anesthesia records revealed significant IH as measured by systolic blood pressure (SBP), diastolic blood pressure (DBP) and MABP recordings (Figures 2-4). Lisinopril and Hydrochlorothiazide were promptly discontinued. Postoperative anemia followed with hemoglobin dropping down to 10.4 g/L from 15.8 g/L. He received more intravenous normal saline for hypotension. Intravenous Furosemide was given for persistent oliguria. Subsequently, his urine output improved. Serum creatinine started to fall (Figure 1). He was discharged from the hospital after 3 days on Amlodipine 10 mg daily and Furosemide 40 mg daily for his blood pressure control. His serum creatinine, about one month after the hip arthroplasty was 0.99 mg/dL, indeed lower than his premorbid levels, with an improved eGFR of 85 ml/min/1.73 sq. m BSA (Figure 1).

### Case II

**Figure 5:**
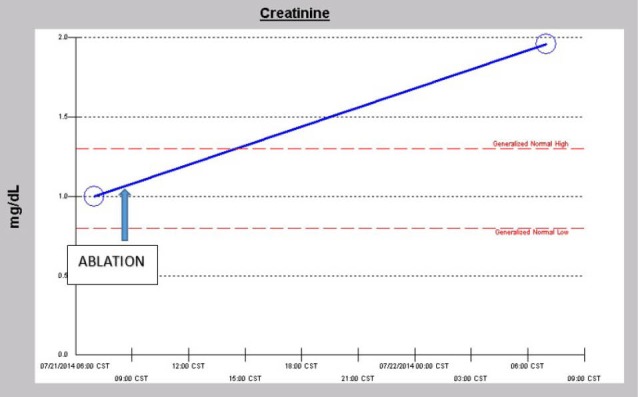
Serum creatinine trajectory showing rapidly rising serum creatinine within 24 hours following the ablation procedure.

**Figure 6:**
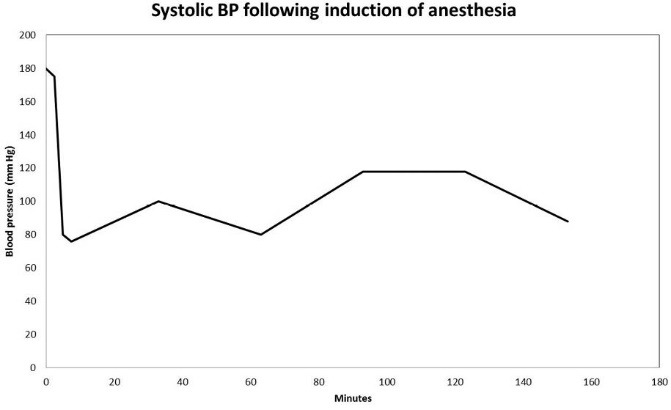
Significant intraoperative hypotension (IH) during anesthesia for the ablation procedure as reflected by continuous systolic blood pressure (SBP) recordings.

**Figure 7:**
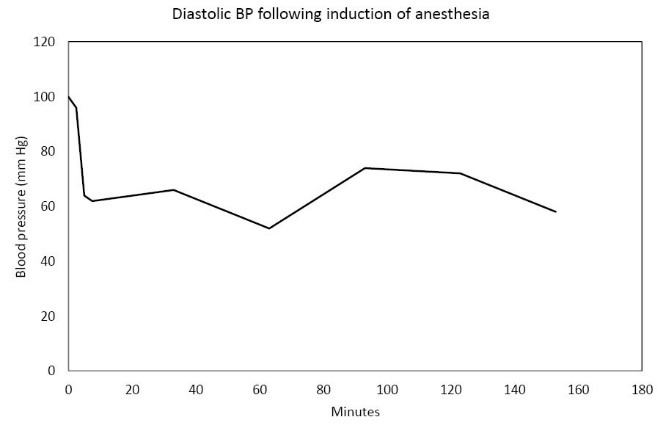
Significant intraoperative hypotension (IH) during anesthesia for right hip arthroplasty as reflected by continuous diastolic blood pressure (DBP) recordings.

**Figure 8:**
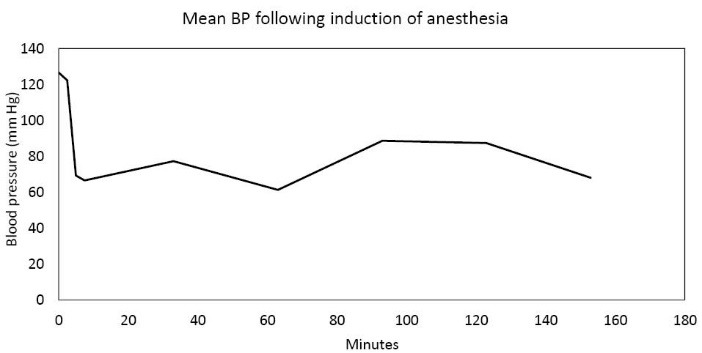
Significant periods of intraoperative hypotension (IH) during anesthesia for the ablation procedure as reflected by continuous mean arterial blood pressure (MABP) recordings.

**Figure 9:**
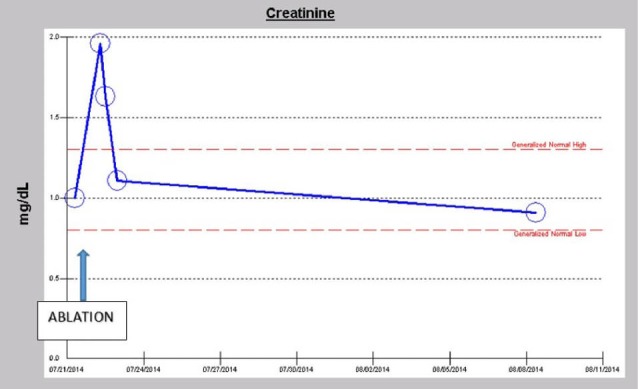
Serum creatinine trajectory up to about two weeks following the elective ablation procedure.

A 57-year-old obese hypertensive type II diabetic Caucasian male patient underwent an elective pulmonary vein isolation with ablation procedure for symptomatic atrial fibrillation early in July 2014. Outpatient medications were chlorthalidone, metformin 1 g BID and lisinopril 40 mg daily. He developed postoperative AKI within 24 hours of the ablation procedure which was notwithstanding described as uneventful (Figure 5). Baseline serum creatinine was 1.0 mg/dL, GFR 81 ml/min/1.73 sq. m BSA, CKD stage II. On postoperative day 1, serum creatinine had increased to 1.96 mg/dL (Figure 5). At this time, he was normotensive and all medical floor BP recordings before and after the procedure were noted to be normal. We had been informed that the procedure in the operating room “went well without complications.” Conversely, our review of the intraoperative anesthesia records revealed significant hypotension during the over 4-hour surgical procedure (Figures 6-8). At nephrology consultation on postoperative day 1, he was nonoliguric and otherwise asymptomatic except for mild lightheadedness. The patient was therefore managed conservatively. Lisinopril and metformin were promptly discontinued. Kidney function subsequently quickly improved. At discharge, with improving kidney function, he had been placed back on Lisinopril with stable kidney function thereafter. His latest serum creatinine later in July 2014 was 0.91 mg/dL, eGFR of 91 mL/min/1.73 sq. m BSA (Figure 9).

## Conclusion

AKI is a relatively common complication of cardiothoracic surgery and has both short- and long-term survival implications, even when AKI does not progress to severe renal failure (27). Given that currently, there are no active effective treatments for AKI, other than renal replacement therapy when indicated, the focus of clinicians ought to be on prevention and risk factor management (27). In a modern series, the incidence of new onset renal failure requiring dialysis was approximately 2% (28). Besides, the pathogenesis of AKI associated with cardiac surgery is complex, poorly understood, and is complicated by the difficulties associated with the acquisition of real-time kidney data in humans (27). This our review and conclusions together with our hypothesis is the first such attempt to achieve real-time kidney data reporting in AKI following both cardiac and non-cardiac surgery (27-29).

## Risk factors for postoperative acute kidney injury

From our recent retrospective analysis of about 50 related surgical cases seen at the Mayo Clinic Health System, Eau Claire, Northwestern Wisconsin, USA, over the last decade or so, we have identified the following risk factors for worsening postoperative AKI in association with IH. Postoperative AKI led to prolonged hospital stay, higher cost of hospitalization, increased patient morbidity and mortality with concurrent increasing requirements for renal replacement therapy (23-26,29-32).

The risk factors are as follows:

1. Older age > 65 years.
2. Higher stage CKD > IIIA, ie, eGFR<45.
3. Concurrent administration of nephrotoxic agents including NSAIDs and angiotensin inhibition with angiotensin converting enzyme inhibitors (ACEIs) and/or angiotensive receptor blockers (ARBs).
4. Preoperative uncontrolled hypertension
5. Obesity with body mass index (BMI) >30-35.

We could not implicate gender nor type of procedure as specific exacerbating risk factors.

Interestingly, of all the above listed 5 exacerbating risk factors, only withdrawal of potentially nephrotoxic agents is a modifiable risk factor for this syndrome. Quicker surgery and therefore shorter operative times is a probable modifiable risk factor.

In addition, from our experiences, we have hypothesized that the elimination of significant IH would significantly reduce the incidence and severity of postoperative AKI.

We are working on setting up a study protocol to investigate the validity of these hypotheses. This move has been further strengthened by our introduction to the utilization of noninvasive real time hemodynamic monitoring of cardiovascular parameters during intraoperative anesthesia which would potentially revolutionize anesthesia monitoring with enhanced safety (33,34).We hope to integrate this innovative innocuous cardiovascular hemodynamic monitoring paradigm in our soon to be developed RCT protocol. Measured outcomes would include patient morbidity indices such as AKI, severity of AKI, need for RRT, length of hospital stay, cost of hospitalization, improved operative outcomes in both cardiac and noncardiac surgery, and would invariably lead to huge savings in healthcare dollars. From our analysis of intraoperative anesthesia blood pressure recordings at the Mayo Clinic Health System, Eau Claire, in Northwestern Wisconsin, we hypothesize that SBP maintenance in the range of 95-105 during HA or CH, more so in the older (>65 years old) with later stage CKD (≥ III CKD or eGFR<60 ml/min/1.73 sq m BSA), will mitigate the occurrence of postoperative AKI. Moreover, it is indeed possible that some of the significant benefits of HA or CH such as reduced blood loss may still be fairly retained at these albeit higher SBP levels.

Preventative medicine is a neglected art in modern day practice of medicine. Prevention is always better than cure. According to Yach and Calitz, (35) “the greatest increase in health care spending between 2000 and 2011 was attributable to drugs, medical devices, and hospital care, with the cost of treating non-communicable diseases (NCDs) estimated to exceed 80% of annual healthcare expenditure, whereas 3% was spent on public health and disease prevention programs.” The National Institutes of Health (NIH) estimates that 20% of its $30 billion annual budget is allocated to prevention; however, less than 10% is spent on human behavioral interventions that target the major modifiable risk factors(35). More investment in prevention science could lead to greater health gains at lower cost (35). We support such reengineering of nephrology practice whereby a lot more emphasis would now be placed on preventative nephrology (30-32,36).

We strongly advocate and submit that our suggested approach, heretofore embraced in this review, to reduce IH while at the same time limiting exposure to potential nephrotoxic agents including angiotensin inhibition, would be far cheaper and more effective in limiting postoperative AKI in our CKD patients than any esoteric pharmaceutical interventions such as the perioperative prophylactic use of such agents as vasodialtors, aspirin, clonidine or fenoldopam to mitigate against cardiac postoperative AKI (37-39). Such attempts in the past in randomized clinical trials have simply been unsuccessful (37-39). Once again, we posit that prevention is better than cure (35).

Finally, this review article has attempted to re-establish the utility of the analysis and critical scrutiny of individual patient-level serum creatinine trajectories in the management of the renal patient (29,40). In 2 recent publications including a book chapter in print, we have revisited the experiences and use of this almost forgotten nephrology methodology in the diagnosis and prognostication of the renal patient by citing various patient experiences from Mayo Clinic Health System, Eau Claire, in Northwestern Wisconsin, and related patient experiences in the Renal Unit, Nnamdi Azikiwe Teaching Hospital, Nnewi, Anambra State, Nigeria (29,40). It remains our hope that these two publications will rekindle renewed interest in the utilization of the individual patient-level serum creatinine trajectories for both real-time management of the renal patient and for short-term, medium-term and long-term renal prognostication purposes (29,40).

## Authors’ contribution

MACO; Conception, design, acquisition of data, data analysis, interpretation of data, drafting the article and final approval of manuscript. NA; Critical revising for important intellectual content, design, final approval of manuscript.

## Conflicts of interest

The authors declared no competing interests.

## Ethical considerations

Ethical issues (including plagiarism, data fabrication, double publication) have been completely observed by the authors.

## Funding/Support

None.
